# Efficacy and safety of diffusing alpha-emitter radiation therapy (DaRT) for head and neck cancer recurrence after radiotherapy

**DOI:** 10.1007/s10147-025-02720-6

**Published:** 2025-02-19

**Authors:** Ryo-ichi Yoshimura, Kazuma Toda, Hiroshi Watanabe, Masahiko Miura, Ryoichi Notake, Naoya Murakami, Hiroshi Igaki, Satoshi Nakamura, Rei Umezawa, Noriyuki Kadoya, Keiichi Jingu, Jun Itami

**Affiliations:** 1 Department of Radiation Therapeutics and Oncology, Institute of Science Tokyo (Tokyo Medical and Dental University), 1-5-45 Yushima, Bunkyo-Ku, Tokyo, 113-8519 Japan; 2 Department of Radiology, Japanese Red Cross Musashino Hospital, Tokyo, Japan; 3 Department of Dental Radiology and Radiation Oncology, Institute of Science Tokyo (Tokyo Medical and Dental University), Tokyo, Japan; 4 Radiology Center, Institute of Science Tokyo Hospital (Tokyo Medical Dental University Hospital), Tokyo, Japan; 5 Department of Radiation Oncology, National Cancer Center Hospital, Tokyo, Japan; 6 Department of Radiation Oncology, Juntendo University Hospital, Tokyo, Japan; 7 Division of Radiation Safety and Quality Assurance, National Cancer Center Hospital, Tokyo, Japan; 8 Department of Radiation Oncology, Tohoku University Graduate School of Medicine, Sendai, Japan; 9 Shin-Matsudo Accuracy Radiation Therapy Center, Shin-Matsudo Central General Hospital, Matsudo, Japan

**Keywords:** Alpha-emitter radiation therapy, Recurrent head and neck cancer, Response rate, Adverse event

## Abstract

**Background:**

To evaluate the efficacy and safety of diffusing alpha-emitter radiation therapy (DaRT) for recurrent head and neck cancer (rHNC) after radiotherapy.

**Methods:**

This study was a multicenter prospective clinical trial. Eligibility criteria included all patients with biopsy-proven rHNC and history of radiotherapy. The efficacy of DaRT was evaluated in terms of tumor shrinkage after 10 weeks of DaRT seed implantation. To assess safety of DaRT, radioactivity levels in blood and urine were measured, and incidence and grade of adverse events (AEs) were evaluated.

**Results:**

Between 2019 and 2021, DaRT was performed in 11 patients and completed in 10 patients with 11 tumors. The tumor sites included the tongue (*n* = 3), buccal mucosa (2), lips (2), floor of the mouth (1), soft palate (1), nose (1), and subcutaneous layer (1). Nine tumors were confirmed to be squamous cell carcinoma, and the remaining two tumors were basal cell carcinoma and neuroblastoma. Complete response (CR) and partial response (PR) were observed in three and six patients, respectively. The response rate was 81.8%. The maximum average radioactivity levels in blood and urine were 42.5 Bq/cm^3^ and 8.4 Bq/cm^3^, respectively, on the second day after implantation. Forty AEs were observed in all 11 patients, including 22 Grade 1 AEs, 16 Grade 2, and 2 Grade 3 (hypertension and seed remnants).

**Conclusion:**

The initial response of rHNC after radiotherapy to DaRT was favorable, and the incidence and grade of AEs were acceptable, as compared to existing therapies.

## Introduction

Radiotherapy (RT) with or without concurrent chemotherapy has been established as the primary treatment for various subsites and stages of head and neck cancer (HNC). However, the incidence of relapse is reported in 15–50% of patients [1, 2]. Although salvage surgery is considered to be the best possible curative treatment for these patients, most recurrences are inoperable, given the infiltrative property of recurrent tumors and the comorbidity or poor performance status of pretreated patients [1]. Re-irradiation is a therapeutic alternative for patients who are not candidates for surgical treatment, but severe toxicity was reported in 18–75% of patients [1, 3]. Chemotherapy with cisplatin or cisplatin in combination with other anticancer agents has been used to treat patients with recurrent and/or metastatic HNC, and response rates have been high with improvement in overall survival compared to best supportive care [4, 5]. Moreover, the combination of chemotherapy (cisplatin and fluorouracil) and monoclonal antibody therapy (cetuximab) significantly improved overall survival with an absolute advantage of 2.6 months in comparison with chemotherapy alone (7.4 months). This combination therapy has become the standard of care for recurrent head and neck cancer (rHNC). However, due to higher incidence of grade 3–4 toxicities (82%), administration of this regimen has not been indicated for all of these patients [4, 6]. Immune checkpoint inhibitors have demonstrated the potential to control cancer by immune activation, but the clinical trials using pembrolizumab or nivolumab for the treatment of patients with recurrent and/or metastatic head and neck squamous cell carcinoma (SCC) showed that the overall response rates were 13–18% and grade 3–4 adverse events (AEs) were reported in 9–15% [4, 7–9]. These results seem to indicate that the response rate for rHNC has been improved, but is still low, and can only be applied to a limited number of patients in light of expected AEs. As a result, many patients with rHNC continue to suffer from various symptoms but have no effective treatment options in practice.

Diffusing alpha-emitter radiation therapy (DaRT) is a novel method for the delivery of alpha particles to solid tumors. The DaRT seed consists of a biocompatible, inert, and metallic scaffold with low activity of ^224^Ra. Once inside the tumor, the seeds are designed to continuously release ^220^Rn atoms by recoil into the tumor tissue. ^220^Rn, a noble gas, diffuses freely as a free atom into surrounding tumor tissues, decaying by alpha emission up to 2–3 mm away from the seed’s surface. This emission is followed by additional alpha emissions by ^216^Po at the same location and by alpha-emitting daughters of ^212^Pb-^212^Bi and ^212^Po. The decay chain continues until the formation of stable ^208^Pb, while ^212^Pb atoms entering the bloodstream are sequestered by red blood cells [10–12]. The therapeutic potential of alpha particles in the treatment of cancer has long been recognized because of its high linear energy transfer and short range, which minimize damage to the surrounding area. It is considered to have a high biologic effect on recurrent tumors after RT. The efficacy of DaRT has been proven in a series of preclinical studies on tumors with different histologic features [11–15].

In this multicenter clinical trial, we evaluated the efficacy and safety of DaRT in patients with rHNC after RT.

## Patients and methods

### Study design and patients

This study was an open-label, single-arm, multicenter, prospective clinical trial. The study was conducted in accordance with the principles of the Declaration of Helsinki, international ethical guidelines of Council for International Organizations of Medical Sciences, and guidelines of Good Clinical Practice. The study protocol, informed consent form, and other relevant documents were approved by the institutional review boards and independent ethics committees of the participating hospitals, including National Cancer Center Hospital, Tohoku University Hospital, and Tokyo Medical and Dental University Hospital. All patients enrolled in this trial provided written informed consent before protocol therapy was initiated.

This study included patients with biopsy-proven rHNC and a history of RT, non-response or non-adherence to medical therapy, and no other treatment options. Eligibility criteria were as follows: patients with a tumor size of ≤ 5 cm in the longest diameter, age of ≥ 18 years with an Eastern Cooperative Oncology Group Performance Status Scale of ≤ 2, a life expectancy of > 6 months, stable vital signs, platelet count of ≥ 100,000/mm^3^, prothrombin time of ≤ 1.8, written informed consent, and target tumor assessable on revised Response Evaluation Criteria in Solid Tumors (RECIST) version 1.1.

Patients were excluded, if they had ongoing treatment with immunosuppressant medications (including corticosteroids), history of allergy to anesthesia or other drugs, metastases requiring treatment, ongoing treatment with chemotherapy, immunotherapy or molecular targeting agents in the past 30 days, treatment with immune checkpoint inhibitors in the past 2 months, enrollment in another clinical trial in the past 30 days, pregnancy or lactation, or unwillingness to sign a consent form.

### Treatment procedure

**Fig. 1:**
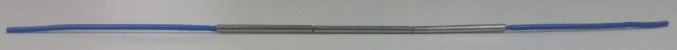
Three DaRT seeds are fixed in suture. One DaRT seed is 0.7 mm in diameter and 1 cm long

The DaRT seeds and applicators to administer them were produced by Alpha Tau Medical, Ltd. (Jerusalem, Israel) and, at the time this article was written, branded in regions outside of Japan as “Alpha DaRT™” technology. Each seed consisted of a 10-mm long and 0.7-mm diameter 316LVM stainless steel tube with ^224^Ra fixed to its surface with a radioactivity of 2 μCi (74 kBq). Up to six seeds were linearly threaded on a single monofilament suture (Fig. 1), sealed in glycerine, and contained within an applicator needle [15].

First, the applicator insertion method and DaRT placement were simulated based on tumor images used for planning. The applicators were inserted into the tumor to geometrically cover the tumor with DaRT seeds at ≤ 5-mm intervals. With this insertion method, tumors could receive a minimal total dose of approximately 10 Gy by alpha-emitter radiation. This technique which results in tumor destruction has been previously demonstrated [11–14]. Planning included maintaining a distance of 10-mm from the major blood vessels. If tumor thickness exceeded 5 mm, multi-layer applicator insertions were planned. The number of applicators and DaRT seeds per applicator were calculated and ordered, as required by the treatment plan.

**Fig. 2:**
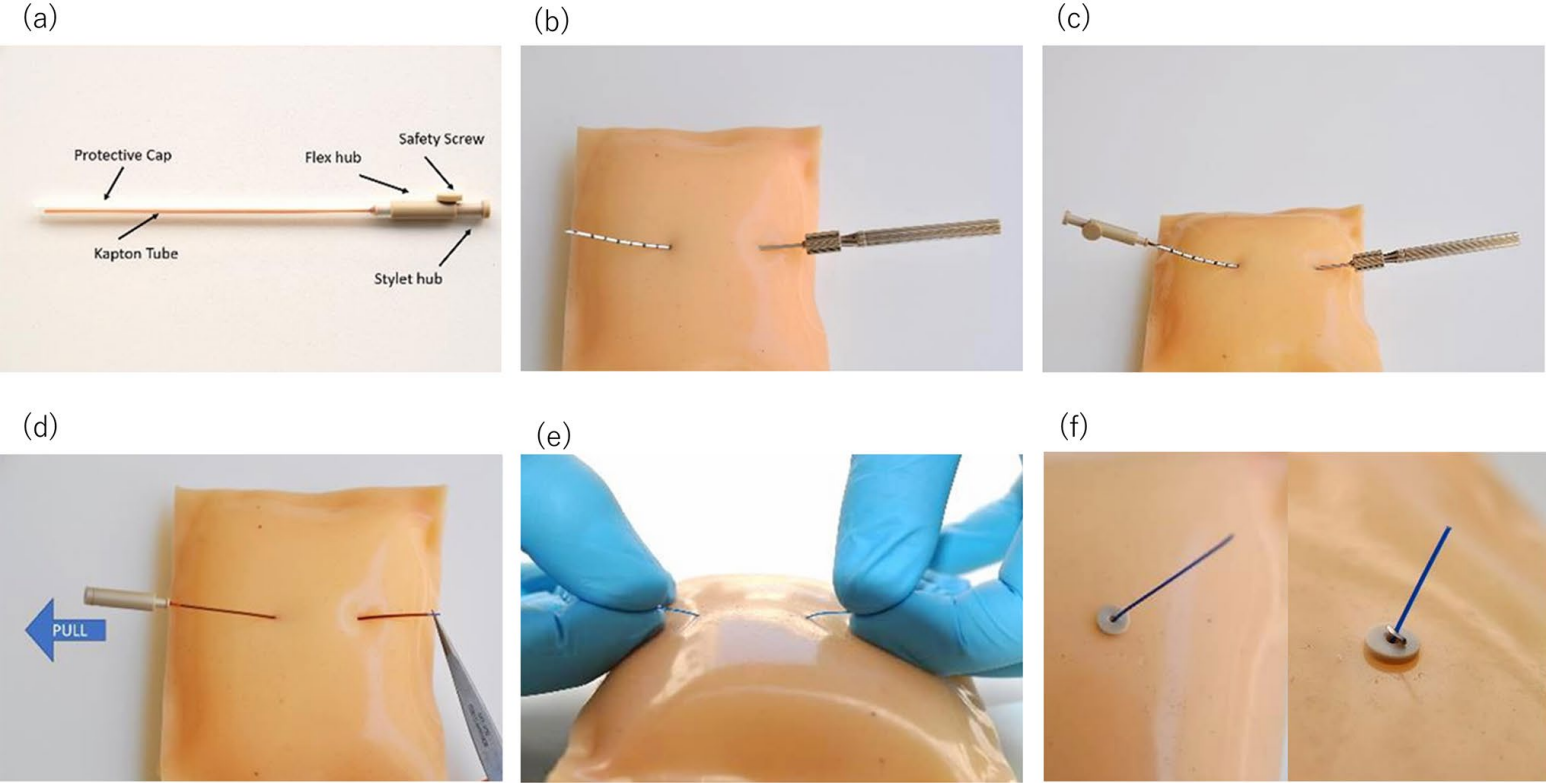
**a** The applicator contains a loaded Kapton flexible tube, stylet, protective cap and safety screw. A suture with fixed DaRT seeds is set inside the Kapton tube. **b** Insert the needle into the skin and through the tumor until the tip of the needle comes out of the other side of the body. **c** Insert the Kapton tube into the needle and remove the safety screw and push the stylet forward. **d** After the suture is well placed, use the forceps to hold it in place and pull the Kapton tube out from the body. **e** If needed, adjust the location of the seeds by pulling the suture from either side. **f** Place buttons and clips on the both sides of the suture, and cut off the leftovers of the suture

According to the pre-treatment plan, DaRT seeds were inserted under local anesthesia at participating institutions on an inpatient basis. The insertion procedure for DaRT is shown in Fig. 2. After DaRT placement, head and neck CT was used to assess final DaRT positions within the tumor for treatment quality assurance. The half-life of the DaRT seed is 3.7 days, and the period of seed implantation was set at 14–21 days to ensure that the lesion is sufficiently irradiated. Seeds were removed during this period at the discretion of the physician in charge based on each lesion and patient’s condition.

### Evaluation

Assuming a threshold response rate of 30% and expected response rate of 70% with DaRT for locally rHNC, the number of lesions for analysis was set at 10. This is per the modified full analysis set (mFAS) method. This method includes only the patients who received treatment.

The primary endpoint of this study was efficacy of DaRT as determined by response rate at 10 weeks from insertion using RECIST criteria ver1.1. Tumor size was measured directly using digital calipers or CT images on day 0, week 1, 2, 3, 4, 10 and 12, and the same measurement method was used on day 0 and week 10 for the same cases to evaluate response.

The secondary endpoint of the study was safety of DaRT. The incidence and grade of AEs in the full analysis set (FAS) were assessed for all patients during the observation period according to the Common Terminology Criteria for Adverse Events (CTCAE) version 5.0/Medical Dictionary for Regulatory Activities (MedDRA) version 20.1. The patients who developed an AE were followed until the AE resolved or their condition stabilized. In addition, radioactivity levels in the blood and urine were measured the day before insertion and on days 2, 4, and 14 after DaRT placement.

These evaluations were performed by the investigators and determined at a central case review meeting.

## Results

### Patient registration

Between June 2019 and December 2021, 15 patients with 16 tumors were enrolled in this study. However, four patients were excluded before treatment. The reasons for exclusion included maximum tumor diameter above 5 cm in one patient, quick progression of tumor growth with possible yield of size beyond the acceptance criteria by the start of treatment in two, and difficulty to insert treatment device (as determined by screening test) in one. The remaining 11 patients were hospitalized, underwent the treatment procedure, and were included in the FAS for safety evaluation. However, one of the patients in the FAS developed severe hypertension due to patient’s anxiety during the implantation procedure, forcing the investigators to halt the implantation midway through the procedure. Therefore, DaRT seed implantation was completed in 10 patients with 11 lesions, and they constituted the mFAS for efficacy evaluation.

The characteristics of the 11 lesions in 10 patients were shown in Table 1. Seven patients had 14 cancers in their previous medical history, and had completed treatment for these previous cancers. The 11 tumor sites treated in this study included the tongue (*n* = 3), buccal mucosa (2), lip (2), floor of the mouth (1), soft palate (1), nose (1), and subcutaneous layer of the head (1), all of which were the same sites as the primary sites. Nine tumors were SCCs, one tumor was a basal cell carcinoma of the nose, and the other was a subcutaneous neuroblastoma. Seven lesions in 6 patients had been irradiated with 48–70 Gy (median, 65 Gy) of external beam radiotherapy (EBRT), which were conventional RT techniques using X-ray or electron beam, or intensity-modulated radiotherapy (IMRT). Four lesions in 4 patients had been irradiated with 60–162 Gy (median, 70 Gy) by brachytherapy. One of these 4 brachytherapy patients also received EBRT for a combined dose of 30 Gy. The mean volume of 11 tumors was 783 mm^3^ (range 141–1920 mm^3^). An average of 25.6 seeds (range: 6–83) were implanted, and the treatment duration was 14 days in all 11 lesions. Follow-up ranged from 82 to 131 days with a median of 84 days.

**Table 1:** Characteristics

Patient/tumor no	Age	Sex	PS	History of cancer	Site of tumor	Histology	Previous treatment	Stage	Tumor size long/short/depth (mm) volume (mm^3^)	No. of seed	Result
1/1	84	M	0	Lung ca Laryngeal ca	Nose	Basal cell carcinoma	RT: Electron 65 Gy	r2	22/21/8 1920	83	PR
2/2	62	M	0	Esophageal ca Gastric ca	Soft palate	SCC	Anticancer agent RT: X-ray 60 Gy	r4	21/10/3 500	13	CR
3/3	66	M	0	None	Head Subcutaneous	Neuroblastoma	Anticancer agent Surgery RT: X-ray 30 Gy, Electron 30 Gy	–	24/18/7 1620	28	SD
4/4	82	F	0	None	Buccal mucosa	SCC	Anticancer agent, RT: Au-BT 67 Gy	r1	20/16/3 753	12	PR
5/5	73	M	0	Lung ca Bladder ca	Tongue	SCC	RT: X-ray 30 Gy Cs-BT 60 Gy Au-BT 85 Gy, 77 Gy	r1	10/6/3 141	6	CR
6/6	45	M	0	None	Tongue	SCC	Anticancer agent Surgery RT: Ir-BT 70 Gy Au-BT 162 Gy	r1	12/10/3 319	12	PD
7/7	72	M	0	Tongue ca	Tongue	SCC	Surgery RT: Ir-BT 70 Gy	r3	30/18/3 1271	30	PR
8/8	81	M	1	Oropharyngeal ca	Buccal mucosa	SCC	Anticancer agent RT: X-ray 65 Gy	r1	12/8/4 201	11	PR
9/9	76	M	1	Esophageal ca Prostate ca Gastric ca	Floor of the mouth	SCC	RT: X-ray 70 Gy	r2	25/15/8 1200	45	CR
10/10	74	F	1	Hypopharyngeal ca Esophageal ca	Lower lip	SCC	Surgery, RT: X-ray 48 Gy	r1	15/8/5 370	20	PR
10/11	Same as above				Upper lip	SCC	Surgery RT: X-ray 48 Gy	r1	20/7/4 320	22	PR

*PS* performance status, *ca*. cancer, *RT* radiotherapy, *Au-BT* brachytherapy using Au-198 grains, *Cs-BT* brachytherapy using Cs-137 needles, *Ir-BT* brachytherapy using Ir-192 hair and single pins

### Radioactivity in blood and urine

Radioactivity levels in blood and urine were evaluated in 10 patients in the mFAS. Outlier levels of radioactivity in blood and urine were reported in each case, and these were considered to be measurement errors and excluded from the evaluation.

The average levels of radioactivity in blood were 34.7 Bq/cm^3^ (maximum, 106 Bq/cm^3^) on day 1, 42.5 Bq/cm^3^ (maximum, 128) on day 2, 34.0 Bq/cm^3^ (maximum, 105) on day 4, and 5.0 Bq/cm^3^ (maximum, 16.9) on day 14. The average levels of radioactivity in urine were 6.6 Bq/cm^3^ (maximum, 16 Bq/cm^3^) on day 1, 8.4 Bq/cm^3^ (maximum, 20) on day 2, 5.1 Bq/cm^3^ (maximum, 14.6) on day 4, and 0.9 Bq/cm^3^ (maximum, 2) on day 14. The radioactivity levels in both blood and urine were highest on day 2, and they decreased to about 10% of those levels on day 14.

**Fig. 3:**
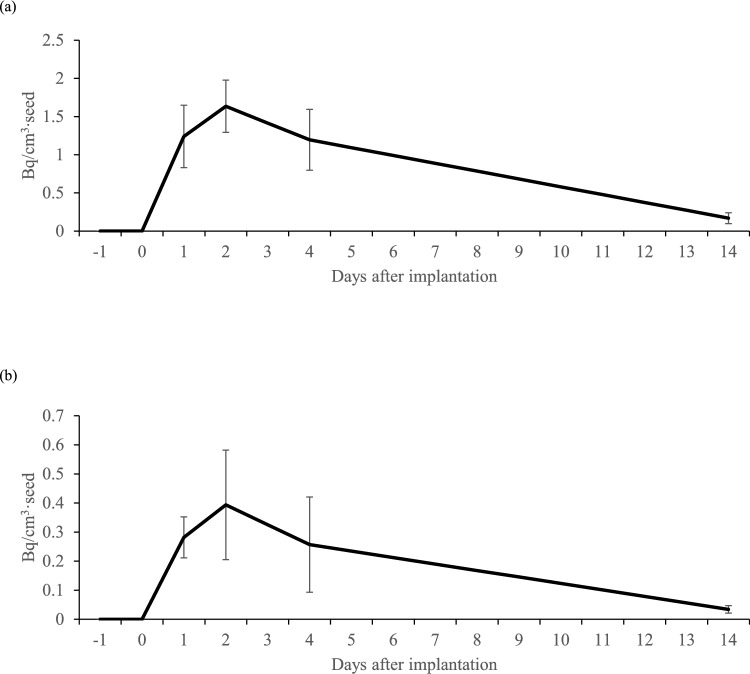
**a** Daily change in blood radioactivity concentration per seed (Mean ± SD); **b** Daily change in urinary radioactivity concentration per seed (Mean ± SD)

A strong correlation existed between the number of seeds and radioactivity level in blood during the entire period, with coefficients of determination (*R*^2^) of 0.96, 0.98, 0.96, and 0.94 on days 1, 2, 4, and 14, respectively. The correlation between the number of seeds and radioactivity level in urine was weaker than that in blood, with *R*^2^ of 0.88, 0.61, 0.18, and 0.93 on days 1, 2, 4, and 14, respectively. Daily changes in radioactivity levels in blood and urine for the equivalent of one implanted seed are shown in Fig. 3a and b.

### Tumor response

**Fig. 4:**
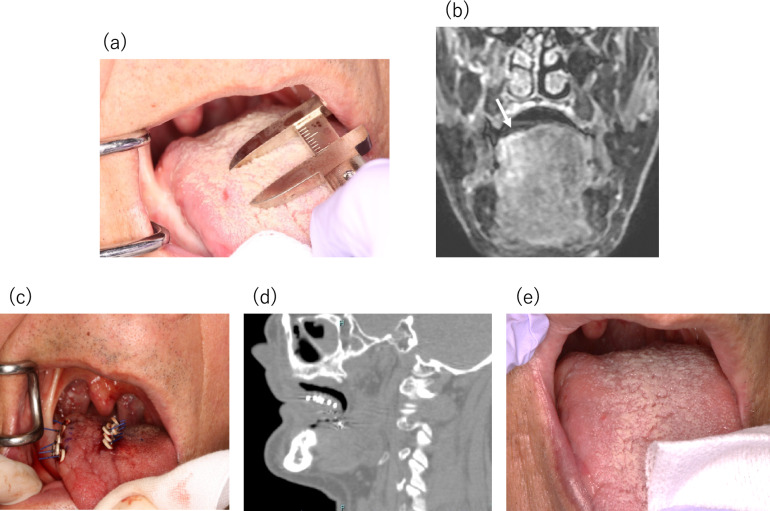
Clinical course of Patient No. 5 with recurrent tongue SCC after brachytherapy treated with DaRT: **a** A tumor before treatment with size of 10 × 6 × 3 mm; **b** MRI coronal section showing tumor (arrow); **c** State of six DaRT seeds implanted in a single plane; **d** Sagittal reconstruction CT image of inserted DaRT seeds; **e** No change was observed until 4 weeks after initiation of treatment, but the tumor disappeared after 10 weeks of treatment

**Fig. 5:**
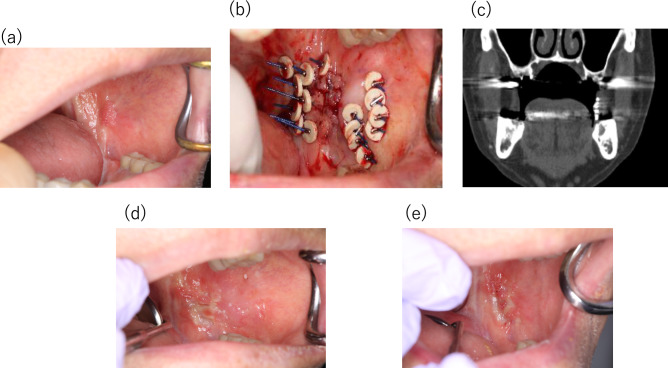
Clinical course of Patient No. 8 with papillary SCC of buccal mucosa after radiotherapy. **a** A tumor before treatment with size of 12 × 8 × 4 mm. **b** State of 11 DaRT seeds implanted in a single plane. **c** Coronal reconstruction CT image of inserted DaRT seeds; **d** Mucositis appeared 1 weeks after seed removal. **e** Mucosal erosion 12 weeks after treatment. Biopsy showed no malignancy

**Fig. 6:**
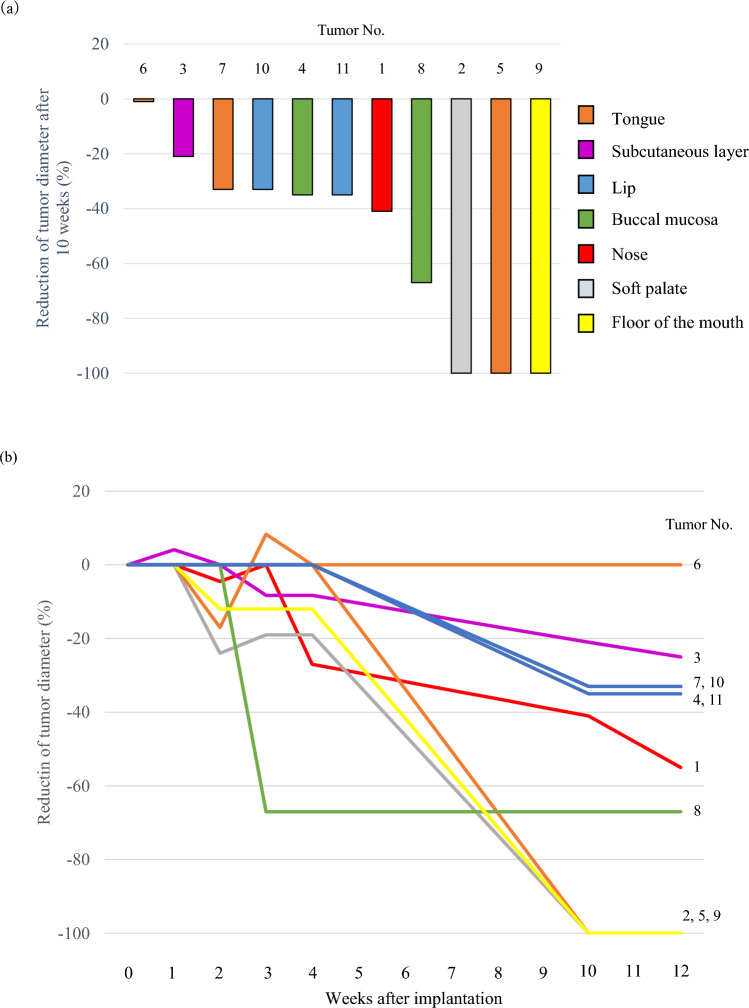
**a** Reduction rate of tumor size after 10 weeks of treatment; **b** Time course of the reduction rate of tumor size

Complete response (CR, Fig. 4), partial response (PR, Fig. 5), stable disease (SD), and progressive disease (PD) were observed in 27.3% (*n* = 3), 54.5% (6), 9.1% (1), and 9.1% (1) of the lesions, respectively (Fig. 6a). The response (CR or PR) rate for all 11 lesions after 10 weeks was 81.8% (95% CI: 48.2–97.7%). Tumor No. 1 and 3 continued to shrink after week 10, but all lesions, including these two, had the same RECIST evaluation at the last observation date (week 12) as at week 10.

The spider plot in Fig. 6b shows the time course of the tumor diameter rate. The average percentage changes were + 0.37% (range 0 to + 4.1) at week 1, – 5.2% (– 24 to 0) at week 2, – 8.9% (– 67 to + 8.3) at week 3, – 12.1% (– 67 to 0) at week 4, – 51.4% (– 100 to 0) at week 10, and – 54.8% (– 100 to 0) at week 12.

The lesion evaluated as SD was a subcutaneous recurrence of olfactory neuroblastoma of the head (Patient No. 3). Its lesion volume was reduced by about 70% (440/1620 mm^3^), but the maximum diameter of the lesion, the baseline, was reduced by only 21% (5/24 mm). Therefore, the tumor was rated as SD. The second lesion, treated in a tongue cancer patient (Patient No. 6), was evaluated as PD. At week 10, the target tumor size was unchanged from baseline, but a new, untreated lesion was found in close proximity, resulting in PD based on assessment criteria.

### AE causally related to DaRT

Altogether, 40 AEs were reported in all 11 patients in the FAS. Among the AEs, 22, 16, and two AEs were Grades 1, 2, and 3, respectively (Table 2). AEs related to application and device insertion were the most common, occurring as 27 AEs in nine patients. However, none of them were Grade 3 or higher. They occurred immediately or early after initiation of treatment and recovered early after the seeds were removed.

**Table 2:** Adverse events in 11 patients

	All grades	Grade 1	Grade 2	Grade 3	Days from implantation to onset range (median)	Days of symptomatic period range (median)
Total	40	22	16	2	0–27 (1)	1–128 (17)
Blood and lymphatic system disorders						
Anemia	1	1	0	0	1	83
Vascular disorders						
Hypertension	2	1	0	1	0	1
Respiratory, thoracic and mediastinal disorders						
Nasal obstruction	2	2	0	0	3, 22	3, 7
Gastrointestinal disorder						
Pharyngodynia	1	0	1	0	1	1
General disorders and administration site conditions						
Application site inflammation	2	0	2	0	1	19, 81
Application site pain	1	1	0	0	0	30
Medical device site abscess	1	1	0	0	11	16
Medical device site erythema	2	2	0	0	1, 14	8, 127
Medical device site hemorrhage	1	1	0	0	0	15
Medical device site edema	1	1	0	0	10	4
Medical device site pain	9	3	6	0	0–20 (0)	1–128 (19)
Medical device site scab	1	1	0	0	6	1
Medical device site swelling	6	5	1	0	0–7 (1)	3–109 (20)
Medical device site ulcer	3	0	3	0	14–27 (14)	15–55 (16)
Pain	1	0	1	0	0	81
Injury, poisoning and procedural complications						
Foreign body	3	1	1	1	14, 14	N.A
Procedural site pain	1	1	0	0	20	7
Radiation mucositis	1	0	1	0	3	17
Investigations						
Creatinine increased	1	1	0	0	7	15

Grade 3 hypertension due to patient anxiety was reported in a 79-year-old woman during the seed implantation procedure; therefore, the procedure had to be halted. The treatment was discontinued, and all implanted seeds were left behind (Grade 2 foreign body) and removed the next day. The remaining seed, a Grade 3 foreign body related to treatment procedure, was observed in Patient No. 7. In addition, a suture remnant, a Grade 1 foreign body, was observed in this patient who had 30 DaRT seeds inserted into his 1271 mm^3^ tumor. At the time of seed removal on day 14, the two seeds remained in the tongue after the suture was pulled out. During the same procedure, in another suture, two seeds detached from the suture and fell off, leaving the suture and clip in the tongue. The two remaining seeds were finally retrieved from the tongue ulcer site on week 12, and the remaining suture and clip were removed at the surgery on week 19.

## Discussion

In this study, 11 patients with rHNC after RT underwent DaRT, and treatments were completed in 10 patients with 11 lesions. The response rate was 81.8% (27.3% CR and 54.5% PR), above the threshold response rate of 30%. Although the incidence of AE was 100% of FAS, Grade 3 was seen in only 18% of patients, and none were Grade 4. The radioactivity level in blood and urine peaked on day 2 and decreased consistently thereafter.

DaRT was shown to be highly effective in treating localized rHNC, which are conventionally difficult to treat. Popovtzer et al. [15] treated 31 locally advanced and recurrent SCC lesions in the skin and head and neck with DaRT and reported a 100% response rate (78.6% CR and 21.4% PR). In addition, D’Andrea et al. [16] treated 10 patients with recurrent or unresectable SCC or basal cell carcinoma of the skin with DaRT and reported a 100% CR.

The timing of evaluating treatment effects differed from study to study. Popovtzer et al. [15] evaluated the effect of DaRT 6 weeks after treatment and reported a good prognosis in the case of CR, whereas D’Andrea et al. [16] reported no relapse in all cases of CR 12 and 24 weeks after treatment, and the 2-year actuarial local recurrence-free survival in a pooled study including patients from those two studies was 77% [17]. In our study, most of SCC lesions showed significant changes from week 4 to week 10, with no changes occurring after week 10. Whereas in non-SCC cases, the tumors continued to shrink after week 10. SCC may respond quickly to DaRT, and evaluation within 10 weeks would be prognostic. However, in our study, the CR rate was lower than those reported by Popovtzer et al. [15] and D’Andrea et al. [16]. Like our study, these studies also used RECIST ver1.1 for the evaluation. The tumors treated in our study were not larger than those in both studies, nor did we implant fewer seeds. The tumor sites reported by Popovtzer et al. were the skin (39%), ear (23%), lip (16%), tongue (10%), nose (6%), and parotid (6%), whereas D’Andrea et al. reported only cutaneous tumors. In our study, 55% of the tumors were observed in the oral cavity, and all 11 lesions previously underwent radiotherapy of approximately 50 Gy or more. Therefore, they were considered to be resistant to radiotherapy, and due to the narrow space in the oral cavity and the hardness of tissues after RT, implantation of seeds in the oral cavity was challenging for us unfamiliar with existing devices. Since a high-dose region of DaRT does not diffuse much beyond 2–3 mm from the seed, the seed spatial arrangement has a strong influence on treatment effect. The CR rate must be lower in the oral cavity than on the other body surfaces because of the difficulty of seed spacing according to treatment plan. Improved insertion techniques and devices could enhance the effectiveness of treatment.

The radioactivity level in blood was proportional to the number of seeds implanted from day 0 to day 14 of DaRT. The highest level of radioactivity was recorded on the second day of treatment (consistent with the buildup of ^212^Pb, as described in [12]). In addition, urine radioactivity peaked on day 2 but did not show a strong proportional relationship with the number of seeds. This finding was due to the fact that radioactivity was measured per 1 cm^3^, and radioactivity level in urine is thought to be related to the total urine volume. The decrease of activity over time after the maximum value at day 2 is consistent with the exponential decay of ^224^Ra (with all its short-lived progeny in secular equilibrium) [12]. Popovtzer et al. [15] reported no measurable radioactivity in the blood and urine 30 days after treatment (consistent with the expectation after ~ 8 half-lives of ^224^Ra).

Re-irradiation is a therapeutic alternative for patients who are not candidates for surgery, but a high incidence (8.7–48%) of severe AEs such as radionecrosis, dysphagia requiring feeding tube placement, trismus, and carotid artery blowout is often reported, even with IMRT, stereotactic body irradiation, and heavy particles [1, 3]. In our study on DaRT for rHNC after RT, 18% (2/11) of the patients had Grade 3 AEs, but they were not caused by alpha RT but by the treatment procedure. Although the follow-up of our study was short, and we could not clarify long-term AEs, Popovtzer et al. [15] and D’Andrea et al. [16] reported no acute grade 3 or higher AEs and no device-related severe AEs. Moreover, they subsequently evaluated up to a median of 14.1 months and reported no late AEs, even in cases of HNC [17]. The AEs experienced in this study led to improvements in the connection between the seed and suture. Future development of treatment procedures and device applicators could further enhance and broaden DaRT’s application in the oral cavity tumor treatment.

In conclusion, this study showed that DaRT achieved good initial response for patients with rHNC after RT, and the incidence and grade of AEs was acceptable.

## References

[1] Svajdova M, Dubinsky P, Kazda T (2021) Radical external beam re-irradiation in the treatment of recurrent head and neck cancer: critical review. Head Neck 43:354–366 32996265 10.1002/hed.26485

[2] Elbers JBW, Al-Mamgani A, van den Brekel MWM et al (2019) Salvage surgery for recurrence after radiotherapy for squamous cell carcinoma of the head and neck. Otolaryngol Head Neck Surg 160:1023–1033 30526317 10.1177/0194599818818443

[3] Dionisi F, Fiorica F, D’Angelo E et al (2019) Organs at risk’s tolerance and dose limits for head and neck cancer reiiradiation: a literature review. Oral Oncol 98:35–47 31536844 10.1016/j.oraloncology.2019.08.017

[4] Guidi A, Codeca C, Ferrari D (2018) Chemotherapy and immunotherapy for recurrent and metastatic head and neck cancer: a systematic review. Med Oncol 35:37 29441454 10.1007/s12032-018-1096-5

[5] The Liverpool Head and Neck Oncology Group (1990) A phase III randomized trial of cistplatinum, methotrextate, cisplatinum + methotrexate and cisplatinum + 5-FU in end stage squamous carcinoma of the head and neck. Br J Cancer 61:311–315 2178667 10.1038/bjc.1990.59 PMC1971415

[6] Vermorken JB, Mesia R, Rivera F et al (2008) Platinum-based chemotherapy plus cetuximab in head and neck cancer. N Engl J Med 359:1116–1127 18784101 10.1056/NEJMoa0802656

[7] Seiwert TY, Burtness B, Mehra R et al (2016) Safety and clinical activity of pembrolizumab for treatment of recurrent or metastatic squamous cell carcinoma of the head and neck (KEYNOTE-012): an open-label, multicentre, phase 1b trial. Lancet Oncol 17(7):956–965 27247226 10.1016/S1470-2045(16)30066-3

[8] Chow LQ, Haddad R, Gupta S et al (2016) Antitumor activity of pembrolizumab in biomarker-unselected patients with recurrent and/or metastatic head and neck squamous cell carcinoma: results from the phase Ib KEYNOTE-012 expansion cohort. J Clin Oncol 34(32):3838–3845 27646946 10.1200/JCO.2016.68.1478 PMC6804896

[9] Ferris RL, Blumenschein G, Fayette JJ et al (2016) Nivolumab for recurrent squamous cell carcinoma of the head and neck. N Engl J Med 375(19):1856–1867 27718784 10.1056/NEJMoa1602252 PMC5564292

[10] Arazi L, Cooks T, Schmidt M et al (2007) Treatment of solid tumors by interstitial release of recoiling short-lived alpha emitters. Phys Med Biol 52:5025–5042 17671351 10.1088/0031-9155/52/16/021

[11] Cooks T, Arazi L, Schmidt M et al (2008) Growth retardation and destruction of experimental squamous cell carcinoma by interstitial radioactive wires releasing diffusing alpha-emitting atoms. Int J Cancer 122:1657–1664 18059026 10.1002/ijc.23268

[12] Arazi L, Cooks T, Schmidt M et al (2010) The treatment of solid tumors by alpha emitters released from (224) Ra-loaded sources-internal dosimetry analysis. Phys Med Biol 55:1203–1218 20124656 10.1088/0031-9155/55/4/020

[13] Horev-Drori G, Cooks T, Bittan H et al (2012) Local control of experimental malignant pancreatic tumors by treatment with a combination of chemotherapy and intratumoral (224) Radium-loaded wires releasing alpha-emitting atoms. Transl Res 159:32–41 22153808 10.1016/j.trsl.2011.08.009

[14] Confino H, Schmidt M, Efrati M et al (2016) Inhibition of mouse breast adenocarcinoma growth by ablation with intratumoral alpha-irradiation combined with inhibitors of immunosuppression and CpG. Cancer Immunol Immunother 65:1149 27495172 10.1007/s00262-016-1878-6 PMC11028980

[15] Popovtzer A, Rosenfeld E, Mizrachi A et al (2020) Initial safety and tumor control results from a first-in-human multicenter prospective trial evaluating a novel alpha-emitting radionuclide for the treatment squamous cell carcinomas of the skin and head and neck. Int J Radiat Oncol Biol Phys 106:571–578 31759075 10.1016/j.ijrobp.2019.10.048

[16] D’Andrea MA, VanderWalde NA, Ballo MT et al (2023) Feasibility and safety of diffusing alpha-emitter radiation therapy for recurrent or unresectable skin cancers. JAMA Netw Open 6(5):e2312824 37166798 10.1001/jamanetworkopen.2023.12824 PMC10176125

[17] Popovtzer A, Mizrachi A, D’Andrea MA et al (2024) Extended follow-up outcomes from pooled prospective studies evaluating efficacy of interstitial alpha radionuclide treatment for skin and head and neck cancers. Cancers 16:2312 39001374 10.3390/cancers16132312 PMC11240433

